# FADS Polymorphism, Omega-3 Fatty Acids and Diabetes Risk: A Systematic Review

**DOI:** 10.3390/nu10060758

**Published:** 2018-06-13

**Authors:** Bárbara Brayner, Gunveen Kaur, Michelle A. Keske, Katherine M. Livingstone

**Affiliations:** 1 Laboratory of Nutritional Biochemistry, Centre of Health Science, Federal University of Rio de Janeiro, Rio de Janeiro 21941-902, Brazil; barbara_vitorinobrayner@hotmail.com; 2 Institute for Physical Activity and Nutrition (IPAN), Deakin University, Geelong 3220, Australia; gunveen.kaur@deakin.edu.au (G.K.); michelle.keske@deakin.edu.au (M.A.K.)

**Keywords:** FADS polymorphism, omega-3 fatty acids, type 2 diabetes

## Abstract

The role of *n-*3 long chain polyunsaturated fatty acids (LC *n-*3 PUFA) in reducing the risk of type 2 diabetes (T2DM) is not well established. The synthesis of LC *n-*3 PUFA requires fatty acid desaturase enzymes, which are encoded by the FADS gene. It is unclear if FADS polymorphism and dietary fatty acid intake can influence plasma or erythrocyte membrane fatty acid profile and thereby the risk of T2DM. Thus, the aim of this systematic review was to assess the current evidence for an effect of FADS polymorphism on T2DM risk and understand its associations with serum/erythrocyte and dietary LC *n-*3 PUFA. A systematic search was performed using PubMed, Embase, Cochrane and Scopus databases. A total of five studies met the inclusion criteria and were included in the present review. This review identified that FADS polymorphism may alter plasma fatty acid composition and play a protective role in the development of T2DM. Serum and erythrocyte LC *n-*3 PUFA levels were not associated with risk of T2DM, while dietary intake of LC *n-*3 PUFA was associated with lower risk of T2DM in one study only. The effect of LC *n-*3 PUFA consumption on associations between FADS polymorphism and T2DM warrants further investigation.

## 1. Introduction

Type 2 diabetes mellitus (T2DM) is a chronic disease that is characterized by an elevation of blood glucose levels (fasting glucose >7 mmol/L or HbA1c >6.5%) [1]. T2DM is often preceded by an insulin resistant state, where the normal biological response to the hormone insulin is impaired and insulin production is disregulated (compensatory hyperinsulinemia) to maintain normoglycemia [2,3]. The prevalence of T2DM and insulin resistance is increasing globally, affecting more than 400 million people worldwide [4]. This is leading to increasing rates of co-morbidities, such as neuropathy, hypertension and cardiovascular disease, and their associated healthcare costs [4]. 

The determinants of T2DM include genetic risk, poor diet and a sedentary lifestyle. It is estimated that 40% of first-degree relatives of patients with T2DM develop this disease, however the incidence in the general population worldwide is approximately 6% [5,6]. Dietary and exercise-based interventions have resulted in delayed progression of T2DM in as many as 50–60% of people with insulin resistance or pre-diabetes [7,8]. Moreover, the amount and quality of fatty acid consumption has been linked to risk of developing T2DM [9].

High intakes of saturated fatty acids and *n-*6 polyunsaturated fatty acids (PUFA) have been linked with impaired glucose tolerance and insulin resistance [9,10]. This is likely to be due to accumulation of excess lipids in liver, muscle and adipose tissue and an increase in pro-inflammatory compounds, such as the eicosanoids prostaglandin E2 and leukotriene B4, which are products of omega-6 fatty acid (arachidonic acid (AA)) [11]. In contrast, long chain omega-3 fatty acids (LC *n-*3 PUFA) such as docosahexaenoic acid (DHA) and eicosapentaenoic acid (EPA) are precursors of anti-inflammatory products, including resolvins, docosatriens and protectins [11,12], which have been shown to improve glucose tolerance and insulin sensitivity [12,13]. In a recent meta-analysis investigating the effect of LC *n-*3 PUFA in T2DM patients, the consumption of *n-*3 fatty acids, especially EPA and DHA, was shown to decrease serum triglyceride levels. In addition, the longer the intervention lasted, the better its effect on glucose control and lipid levels [14]. Given the association between LC *n-*3 PUFA and improved insulin sensitivity, it is important to understand if this translates to a reduced risk of developing T2DM. Foods and nutrients are not consumed in isolation, making it important to consider the role *n-*3 fatty acid intakes play within the context of the overall diet, i.e., dietary patterns [15]. Studies have shown that dietary patterns high in oily fish consumption have been linked to lower risk of T2DM [16], yet the impact of these dietary patterns on associations between the FADS polymorphism, plasma LC *n-*3 PUFA concentrations and risk of developing T2DM is unclear. In addition, little is known about how endogenous LC *n-*3 PUFA production and genetic risk influence these relationships.

**Figure 1:**
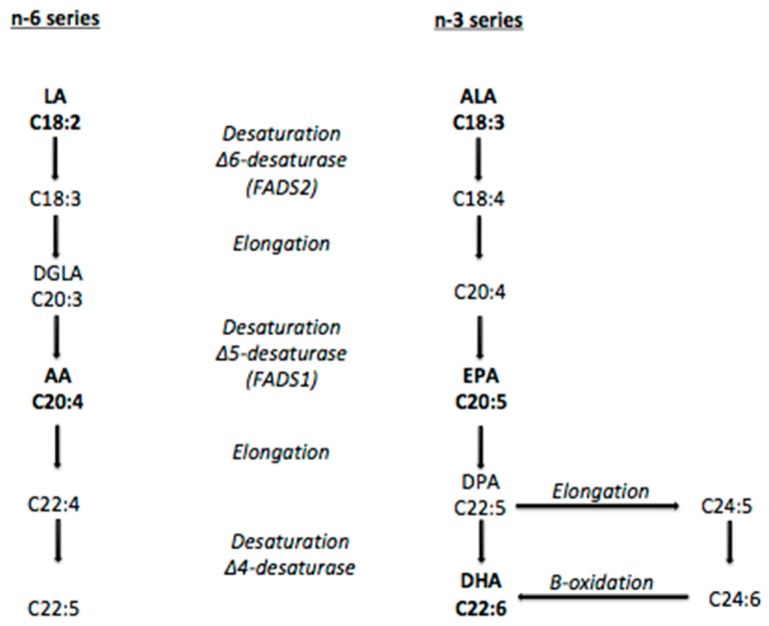
Pathway of desaturation and elongation of *n-*3 and *n-*6 fatty acids. The enzymes Δ6 and Δ5 desaturase are encoded by FADS2 and FADS1, respectively. LA: linoleic acid; DGLA: dihomo-gamma linolenic acid; AA: arachidonic acid; ALA: alpha-linolenic acid; EPA: eicosapentaenoic acid; DPA: docosapentaenoic acid; DHA: docosahexaenoic acid.

The concentration of LC *n-*3 PUFA in red blood cells and plasma is dependent on both dietary intake and adequate endogenous production of these fatty acids [17]. LC *n-*3 PUFA can be endogenously synthesized via metabolism of the essential fatty acid alpha-linolenic acid (ALA). This endogenous production is mediated by the enzymes delta-5-desaturase (D5D) and delta-6-desaturase (D6D), which are encoded by the genes fatty acid desaturase 1 (FADS1) and fatty acid desaturase 2 (FADS2), respectively [18,19] (Figure 1). 

Single nucleotide polymorphisms (SNP) in the FADS gene have been linked to variations in fatty acid composition in various human compartments, such as erythrocyte membrane, plasma and breast milk [20,21,22]. However, little is known about which SNPs are responsible for these alterations [23]. A genetic variation in the FADS gene is linked to lower expression and activity of D5D and D6D, thereby increasing concentrations of the precursors linoleic acid (LA) and ALA but not of their downstream fatty acids AA, EPA and DHA [24,25,26]. The impact of dietary intakes and its potential to attenuate differences between major and minor allele carriers of the FADS polymorphism remains unclear [26,27]. 

Few studies have investigated whether LC *n-*3 PUFA intake is able to mitigate differences in plasma fatty acid profile among carriers of the FADS minor allele. Moreover, very little is known about how the FADS polymorphism and plasma concentrations and dietary intakes of *n-*3 fatty acids or dietary patterns high in *n-*3 fatty acids interact to influence an individual’s risk of T2DM. The aim of this review was thus to systematically evaluate evidence on associations between the FADS polymorphism, plasma LC *n-*3 PUFA concentrations and risk of developing T2DM and understand the role of dietary fatty acid intakes on these associations.

## 2. Materials and Methods 

### 2.1. Study Selection

This review includes publications from human observational studies and randomized controlled trials. Animal and in vitro studies were excluded. In order to be included in this review, the studies were required to include information on (i) FADS polymorphisms; (ii) omega-3 fatty acid intakes; (iii) plasma or erythrocyte membrane omega-3 fatty acid concentrations and (iv) whether participants presented with or were at risk of T2DM. Only publications in English were considered. 

### 2.2. Search Strategy

Published studies between inception and February 2018 were identified from a literature search of four electronic databases: PubMed, Embase, Scopus and Cochrane Library. A manual search of the reference lists of relevant articles was also conducted to identify any additional papers that were not returned by the initial search. The search strategy involved combining three search themes using the Boolean operator ‘and’. The first theme was (‘FADS’ OR ‘Fatty acid desaturase’), the second theme was (‘fatty acid*’ OR ‘*n-*3′ OR ‘*n-*3 fatty acid*’ OR ‘alpha linolenic acid’ OR ‘ala’ OR ‘eicosapentaenoic acid’ OR ‘epa’ OR ‘docosahexaenoic acid’ OR ‘dha’ OR ‘docosapentaenoic acid’ OR ‘dpa’ OR ‘long chain fatty acid*’ OR ‘diet’ OR ‘dietary pattern*’ OR ‘dietary fat*’) and the third theme was (‘type 2 diabetes’ OR ‘pre diabetes’ OR ‘insulin resistance’ OR ‘impaired glucose tolerance’ OR ‘glucose intolerance’). The search results were exported to a Reference Manager software, and were saved in a master file. Duplicates were removed via an in-built function within the software. A detailed record of all stages of the protocol was kept. This systematic review was undertaken in accordance with PRISMA guidelines and has been registered with PROSPERO, the International Prospective Register of Systematic Reviews (registration number: CRD42018084831).

### 2.3. Study Selection and Screening

Two reviewers independently assessed the article titles and abstracts for eligibility according to the inclusion and exclusion criteria. If both reviewers deemed the study suitable, the full text was retrieved for further evaluation. If there was disagreement, a third independent reviewer was used.

### 2.4. Data Extraction and Quality Assessment

Data extraction was performed by one reviewer using a standardized excel form developed by the researchers. A second reviewer checked the extraction for accuracy and consistency. The following information was extracted: (i) intervention characteristics: study design, sample size and country (ii) participant characteristics: age and sex (iii) FADS polymorphism (iv) fatty acids intakes and concentrations: dietary fatty acid intakes (saturated fatty acids, monounsaturated fatty acids, polyunsaturated fatty acids, eicosapentaenoic acid, docosapentaenoic acid and docosahexaenoic acid) and plasma or erythrocyte membrane *n-*3 fatty acid concentrations and (v) T2DM risk: defined by blood glucose levels; HbA1c levels; glucose tolerance; insulin sensitivity or type 2 diabetes. Two independent reviewers assessed the quality of the studies using the Cochrane Risk of Bias Tool [28]. A third reviewer was consulted if there was a discrepancy. The quality of each study was assessed according to the following criteria: measurement protocols, blinding, incomplete data outcome and selective reporting.

## 3. Results

**Figure 2:**
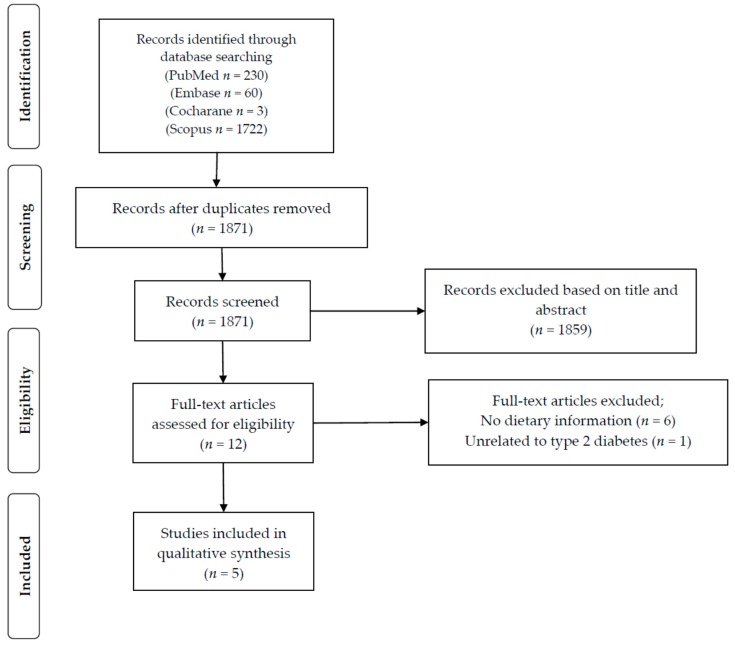
Study selection for inclusion in the systematic review based on the Preferred Reporting Items for Systematic Reviews and Meta-analyses (PRISMA) statement.

The initial search identified a total of 2015 potential studies. After removal of duplicates, titles and abstracts of 1871 papers were screened. Based on this screening process, 1859 articles were excluded for not meeting our pre-defined inclusion criteria. The 12 articles selected after the screening process were then assessed in more depth using the full-text article. As detailed in Figure 2, six of those 12 articles did not have information on dietary intake and were therefore excluded from this review. One article did not report information on type 2 diabetes and was thus also excluded. In total, five articles were deemed eligible and were included in the present review. 

The characteristics of the studies included in this review are presented in Table 1. The first study had a cross-sectional design [29], the second was a prospective cohort [30], the third was a randomized controlled trial [31], the fourth was a case-control study and the fifth was a prospective cohort study [32]. Most of the studies included both male and female participants, except for the cross-sectional study, which only included men [29]. The sample size ranged from 208 [31] to 2114 [30]. All of the studies were conducted with adult participants and the mean age ranged from 31 [31] to 63 years [33]. Three studies investigated the risk of developing T2DM: one by physician diagnosis during the cohort study (using International Classification of Diseases criteria) [30], one by past physician diagnosis (using World Health Organization criteria) [33] and the last one using an oral glucose tolerance test [32]. Two studies [29,31] analyzed fasting glucose and insulin and from that calculated insulin resistance/insulin sensitivity by the homeostasis model assessment (HOMA). All five studies investigated polymorphisms in the FADS gene cluster. Three studies collected information on serum fatty acid composition [29,32,33] and two of those assessed the desaturase enzymes activity [32,33]. One study analyzed erythrocyte membrane fatty acid composition and also investigated desaturase enzymes activity [30]. Three studies used food frequency questionnaires to collect dietary information [30,31,33], one used a 3-day food record [33] and the other used three 24-h recalls, which assessed intake on two typical week days and one atypical day (weekend or holiday) [29]. Dietary intakes reported included intakes of key nutrients and select food groups only. No studies reported overall dietary patterns. 

The majority of the studies included in this systematic review were considered to have low risk of bias in their measurement protocols, blinding of volunteers and personnel, outcomes and selective reporting. Only one study [33] did not provide clear information on the blinding protocol for the participants (performance bias) and the outcome assessment (detection bias) (see supplementary Table S1). 

Kim et al. [29] investigated cross-sectional associations between FADS gene polymorphism (SNPs rs174537, rs174575, rs1000778) and insulin resistance as well as serum fatty acid composition. Findings showed that HOMA-IR was higher in carriers of the minor FADS allele when individuals had higher serum concentrations of DGLA (≥1.4% in total serum phospholipids (*p* for interaction = 0.009) or AA (≥4.6% in total serum phospholipids, *p* for interaction = 0.047). No significant association was found between *n-*3 fatty acid levels in serum phospholipids, FADS polymorphism and HOMA-IR. Regarding dietary lipid intake, no significant association was found between different FADS polymorphisms. Additionally, individuals with this polymorphism had significantly higher fasting insulin (mean 9.7 ± 5.9 µIU/mL) than individuals who were homozygous for the major allele (mean 8.7 ± 3.8 µIU/mL) (*p* < 0.05) [29]. 

In a prospective cohort, Kroger et al. [30] identified that the fatty acid profiles of erythrocyte membrane phospholipids and the activity of desaturase enzymes, but not dietary fatty acids, were strongly linked to the incidence of T2DM. Results showed that high proportions of LA in erythrocyte membrane fatty acid were linked with lower risk of developing T2DM (relative risk (RR) for the highest versus the lowest quintiles of LA concentrations = 0.8 (95% CI: 0.5, 1.1)). In contrast, high proportions of gamma-linolenic acid (18:3*n-*6) and DGLA (20:3*n-*6) predicted increased risk of T2DM (RR for the highest versus the lowest quintiles of gamma-linolenic acid = 2.0 (95% CI: 1.4, 2.9); 1.72 (95% CI: 1.2, 2.5), respectively). The concentration of *n-*3 PUFA was not significantly associated with risk of T2DM development. Furthermore, lower activity of D6D enzyme predicted lower risk of T2DM in carriers of the minor FADS allele (SNP rs174546), compared to individuals without (RR for individuals homozygous for the minor allele TT genotype = 0.60 (95% CI: 0.4, 0.9) vs. heterozygous for the CT genotype = 0.75 (95% CI: 0.6, 1.0)). Dietary fatty acid intake was not significantly associated with T2DM incidence in this study [30]. 

In a randomized controlled trial, Cormier et al. [31] demonstrated that the SNP rs482548 had an interaction effect on the relationship between LC *n-*3 PUFA supplementation and fasting glucose levels. This interaction effect led to higher levels of fasting glucose after supplementation in carrier of the FADS polymorphism (*p* interaction = 0.008). In addition, several SNPs were associated with decreased HOMA-IR in response to LC *n-*3 PUFA supplementation (rs7394871 *p* = 0.03; rs174602 *p* = 0.01; rs174570 *p* = 0.03; rs7482316 *p* = 0.05). 

Yao et al. [33] identified that minor allele carriers of the SNP rs174616 were associated with decreased risk of T2DM in a case-control study. Despite other investigated SNPs not being associated with T2DM development, they were associated with serum PUFA composition; individuals who were carriers of the minor allele had higher serum PUFA and lower LC-PUFA composition. T2DM patients, who were carriers of the minor allele of rs174545 and rs2072114, had lower levels of EPA (*p* = 0.000; *p* = 0.002) and DPA (*p* = 0.006; *p* = 0.0024), respectively. For the SNP rs2072114 concentration of LA was also higher in carriers of the minor allele (*p* = 0.004). The minor allele of rs175602 was associated with lower concentrations of EPA (*p* = 0.007) in T2DM individuals. In addition, desaturase activity of D5D, measured by EPA/ALA ratio, was lower (*p =* 0.009), while D6D, measured by AA/LA ratio, was higher (*p* < 0.001) in T2DM individuals. Moreover, dietary saturated fatty acid intake (*p* < 0.0014) was higher in T2DM cases, whilst PUFA intake was lower (*p* > 0.054) [33]. 

Takkunen et al. [32] demonstrated that total serum LC *n-*3 PUFA concentration (*p* = 0.001) and D5D activity (*p* = 0.011) were associated with lower incidence of T2DM in a prospective cohort. In addition, serum concentrations of EPA (*p =* 0.016) and DPA (*p =* 0.024) were positively associated with insulin sensitivity.

## 4. Discussion

This is the first review to systematically evaluate the evidence regarding the association between FADS polymorphism, plasma or erythrocyte membrane LC *n-*3 PUFA fatty acid concentrations and T2DM risk, and if those relationships are influenced by dietary fatty acid intakes. After conducting a systematic search across four databases, five articles were identified that were eligible for inclusion in this review. The main findings were that FADS polymorphism and higher D5D and lower D6D activity may alter plasma and erythrocyte fatty acid composition, thereby playing a protective role in the development of T2DM. While two studies showed no association between serum *n-*3 PUFA concentration [29] or erythrocyte *n-*3 PUFA concentration [30] and risk of T2DM, these studies did show that higher concentrations of some of the *n-*6 PUFA in serum/erythrocytes (DGLA, 18:3*n-*6 and 20:3*n-*6) were associated with higher risk of T2DM, while 18:2*n-*6 was associated with lower risk of T2DM. In addition, dietary consumption of LC *n-*3 PUFA had a protective association with T2DM in one study [32], but no association was observed in others [29,30,33]. Supplementation with high doses of LC *n-*3 PUFA improved HOMA-IR in some variants of FADS polymorphism but increased fasting glucose levels in carriers of the minor allele for rs482548 (FADS2) [31].

The literature suggests there is an association between serum fatty acid composition and FADS SNPs. The cross-sectional study by Kim et al. [29], conducted with 576 Korean men, showed that the serum fatty acid composition varied in individuals with different SNPs of FADS gene. However, these variations were only significant for *n-*6 fatty acids (18:2*n-*6, AA and DGLA levels in serum phospholipids). Another study investigated this correlation in Caucasian individuals in Germany, with a sample of 727 subjects [20]. The SNPs investigated in this study explained 28% of the variance in AA in the individuals with polymorphism and 12% of its fatty acids precursors. In this study, LC *n-*3 PUFA concentrations also varied between different genotypes: DPA and EPA were lower whilst its precursor ALA was higher in individuals with a polymorphism in the FADS gene [20], suggesting that FADS polymorphism does influence fatty acids levels in blood. Similarly, Malerba et al. [21], genotyped 658 Italian subjects from the Verona Heart Project and measured fatty acid composition not only in serum but also in erythrocyte membrane. This study confirmed that the substrates of D5D and D6D (LA, ALA) were higher in serum and erythrocyte membranes of minor allele carriers, whilst their products (AA, EPA, DPA) were lower. 

Studies have also investigated the relationship directly between the activity of desaturase enzymes and T2DM [30,33]. A prospective cohort of 2114 subjects from Germany investigated associations between dietary fatty acid intakes, T2DM risk and desaturase activity. Higher proportion of LA in erythrocytes predicted lower risk of T2DM development, whilst higher proportions of GLA and DGLA were associated with a higher risk of T2DM development. Additionally, in individuals with this polymorphism, lower D6D activity was related to lower T2DM incidence [30]. Furthermore, a study evaluating the influence of FADS1 and FADS2 genetic variants on desaturase activity and lipid concentrations in 820 T2DM patients identified that FADS1 rs174547 and FADS2 rs2727270 genotypes were significantly correlated to lower levels of D5D and D6D activity in T2DM patients [34]. Another prospective cohort carried out in 407 subjects from Finland found that total serum LC *n-*3 PUFA, proportions of marine *n-*3 FA and the estimated activity of D5D predicted lower incidence of T2DM, which is likely to be due to higher insulin sensitivity [32]. Therefore, it is likely that the FADS polymorphism, which influences D5D and D6D activity, may modulate the risk of developing T2DM. 

Our findings suggest that serum and erythrocyte fatty acid composition may be affected by dietary intake of *n-*3 PUFA. Similarly, there is some research suggesting that the consumption of *n-*3 PUFA may have a beneficial effect on glycemic control and insulin sensitivity [12,13,14], however, the effect of *n-*3 PUFA on risk of T2DM is still unclear. A meta-analysis of prospective studies that focused on dietary *n-*3 PUFA sources, biomarker levels of *n-*3 PUFA and the incidence of T2DM, concluded that the evidence was mixed [35]. Only dietary intake of ALA from plant-based food was found to be modestly associated with lower risk of developing T2DM [35]. The relationship between *n-*3 PUFA intake and T2DM is further complicated when considering the role of FADS polymorphism. Cormier et al. [31] identified that the FADS SNPs were significantly associated with glycemic control, including lower HOMA-IR in response to the fish oil supplementation in Canadian subjects. These findings suggest that LC *n-*3 PUFA supplementation may play a protective role against T2DM in carriers of the minor allele for FADS gene. These findings are consistent with Yao et al. [31] which observed that Chinese individuals who were carriers of the minor allele of the FADS SNP (rs174616) had a lower risk of developing T2DM. Furthermore, higher intake of PUFA appeared to have had a protective effect on this relationship [33]. The protective role of dietary LC *n-*3 PUFA in the development of T2DM has been observed more often in Asian populations than in European/North Americans. Besides cultural differences in the preparation of foods that are rich sources of LC *n-*3 PUFA (raw/steamed vs. deep-fried), genetic factors are likely to strongly influence this association [36].

The mechanism as to how FADS polymorphism and dietary LC *n-*3 PUFA intake affects T2DM development remains unclear [29,31]. However, a possible explanation is that this genetic variant has been linked to lower D6D activity and lower concentrations of AA in plasma, red blood cells and adipose tissue [18,20,22]. This compound is the precursor of pro-inflammatory metabolites, which are related to an increase in overall inflammatory state [11]. Importantly, LC *n-*3 PUFA dietary intake is associated with the production of anti-inflammatory compounds [12] and the FADS polymorphism may lead to lower AA levels in plasma and red blood cells. As a result, this may lead to greater availability of cyclooxygenase and lipoxygenase enzymes to metabolize LC *n-*3 PUFA into their anti-inflammatory metabolites, thereby lowering inflammation and having a positive effect on T2DM risk. While this mechanism is plausible, further clinical research is needed to better understand this potential mechanism of action. In addition, dietary information collected in these studies focused on nutrient intakes only; the role *n-*3 fatty acid intake within the context of dietary patterns remains unclear.

## 5. Conclusions

This review was the first to systematically evaluate the role of FADS polymorphism on *n-*3 fatty acid concentration in plasma or erythrocyte membrane and on T2DM risk, and to identify if those relationships could be influenced by dietary intake. Given the heterogeneity in the study designs and the small number of studies eligible for inclusion in this review, our ability to draw firm conclusions was limited. Nonetheless, this review identified that the FADS polymorphism may influence plasma and erythrocyte fatty acid composition as well as T2DM risk markers, such as HOMA-IR and fasting glucose. All five studies demonstrated that there was a significant positive association between carrying the FADS polymorphism and T2DM risk. However, dietary LC *n-*3 PUFA intake was only associated with lower T2DM risk in one study. When considering which FADS SNPs are involved in these associations, the majority of the studies investigated different SNPs and therefore it was not possible to identify the role of any single SNP on risk of T2DM. Future research, preferably randomized controlled trials, is necessary to understand the mediation effect of dietary fatty acid intake on associations between FADS polymorphism, plasma or erythrocyte fatty acid and risk of developing T2DM. In addition, with an increasing focus on understanding diet as whole, the role of dietary patterns on these relationships warrants further investigation.

## Figures and Tables

**Table 1:** Characteristics of the five studies that met the inclusion criteria of this systematic review.

Author, Year	Country, Age (Mean, SD)	*n*	Study Design	Exposure	Outcome	Results	Conclusion
Kim et al., 2011 [29]	Korea, 30–69 years (48.7, 9.3)	576	Cross-sectional study	FADS polymorphisms: rs174537, rs174575, rs1000778	T2DM risk: fasting glucose (mg/dL), fasting insulin (µIU/mL) and HOMA–IR Fatty acid concentration: serum phospholipid FA composition (relative %): SFA, MUFA, PUFA (ALA, LA, AA, EPA, DGLA, DPA, DHA) Insulin Resistance (HOMA)	HOMA-IR and serum FA composition or FA ratios were associated with the FADS SNPs (rs174575 minor allele carriers had higher HOMA-IR when they had higher concentrations of DGLA (≥1.4% in total FA, *p* for interaction = 0.009) or of AA (≥4.6%, *p* for interaction = 0.047)). No significant association was found between *n-*3 FA serum composition, FADS polymorphism and HOMA-IR.	FADS SNPs were associated with higher HOMA-IR, when individuals had higher serum LC *n-*6 PUFA. No significant association was found for serum *n-*3 FA.
Kroger et al., 2011 [30]	Germany, 35–65 years (Controls 50, 8.9 and cases 55.1, 7.4)	Controls: 2114; cases: 673	Prospective cohort, 7 year follow up	Dietary intake: SFA (% of total fat intake), MUFA (% of total fat intake), *n-*3 PUFA (% of total fat intake), *n-*6 PUFA (% of total fat intake), total PUFA (% of total fat intake) FADS polymorphism: rs174546 Desaturase activity Fatty acid concentration: erythrocyte membrane phospholipids FA (% of total FA), AA/DGLA (D5D) and ALA/LA (D6D) ratios	T2DM risk: clinical diagnosis of T2DM	Risk of T2DM differed according to FA profile of erythrocyte membrane phospholipids. Higher proportions of LA were associated with lower risk of T2DM (RR for extreme quintiles = 0.76 (95% CI: 0.54, 1.08)); whereas higher proportions of GLA and DGLA predicted increased T2DM risk (RR for extreme quintiles = 2.00 (95% CI: 1.38, 2.88); RR = 1.72 (95% CI: 1.18, 2.53), respectively). Activity of desaturase enzymes was linked to incidence of T2DM. Lower activity of D6D predicted lower risk of T2DM in carriers of the minor allele compared to those without (RR CT genotype = 0.75 (95% CI: 0.59, 0.96); TT genotype = 0.64 (95% CI: 0.43, 0.94)). Dietary FA intake was not associated with T2DM.	T2DM incidence was higher in individuals with higher proportions of LC *n-*6 PUFA and lower D6D activity. No association was found with dietary *n-*3 FA intake.
Cormier et al., 2013 [31]	Canada, 18–50 years (30.8, 8.7)	208	Randomized controlled trial, 6 week duration	Dietary intake: 3–3.3 g/day of fish oil (1.9–2.2 g of EPA + 1.1 of DHA) FADS polymorphism: rs174556, rs174627, rs482548, rs2072114, rs12807005, rs174448, rs2845573, rs7394871, rs7942717, rs7482316, rs174602, rs498793, rs174546, rs174570, rs174579, rs174611, rs174616, rs968567	Fasting Glucose (mM), Fasting Insulin (ρ/L), HOMA-IR	SNPs involved in the FADS gene cluster were associated with glycemic control parameters (a supplementation * genotype interaction effect on FG levels was observed for rs482548 (*p* = 0.008)), mainly decreased HOMA-IR, in response to a high dose intervention with *n-*3 PUFA (supplementation * genotype interaction effect for rs174602 (*p* = 0.01); rs174570 (*p* = 0.03); rs7394871 (*p* = 0.03))	FADS SNPs were associated with lower HOMA-IR and higher fasting glucose in response to a high dose *n-*3 PUFA supplementation.
Yao et al., 2015 [33]	China, (Controls: 51.38, 11.27 years; cases: 63.24, 10.43 years)	Controls: 421; cases: 331	Case-control study	Dietary intake: total fat (%E), SFA (%E), MUFA (%E), PUFA (%E) FADS polymorphism: rs174545, rs2072114, rs174602, rs174616 Desaturase activity Fatty acid concentration: serum PUFA composition (% of total FA), EPA/ALA (D5D) and AA/LA (D6D) ratios	T2DM risk *	Minor allele carriers of the rs174616 are associated with a decreased risk of T2DM (*p* = 0.023). Desaturase activity of D5D was decreased (*p =* 0.009), while D6D was increased (*p* < 0.001) in T2DM individuals. Dietary saturated fatty acid (*p* < 0.0014) was significantly higher in T2DM cases, whilst PUFA intake was lower (*p* > 0.054)	Higher PUFAs intake, desaturase activity and SNP rs174616 are associated with decreased risk of T2DM.
Takkunen et al., 2016 [32]	Finland, 40–65 years (55.4, 7.14)	407	Prospective cohort, 6 year follow up	Dietary intake: total fat (g/day), SFA (g/100 g of total fat), MUFA (g/100 g of total fat), PUFA (g/100 g of total fat) Desaturase activity: AA/DGLA (D5D) and ALA/LA (D6D) ratios using FADS polymorphism (rs174550) for validation Fatty acid concentration: 20 serum FA (mol%)	T2DM incidence OGTT (mmol/L)	Total serum LC *n-*3 PUFA (*p* = 0.001) and estimated D5D activity (*p* = 0.011) predicted lower incidence of T2DM.	Lower incidence of T2DM was associated with serum LC *n-*3 PUFA, proportions of marine *n-*3 FA and estimated D5D activity.

FADS: fatty acid desaturase; HOMA: homeostasis model assessment; OGTT: oral glucose tolerance test; FG: fasting glucose; FA: fatty acids; SNP: single nucleotide polymorphism; T2DM: type 2 diabetes mellitus; AA: arachidonic acid; ALA: alpha-linolenic acid; LA: linoleic acid; DGLA: dihomo gamma-linolenic acid; E: energy intake; EPA: eicosapentaenoic acid; DHA: docosahexaenoic acid; GLA: gamma-linolenic acid; D5D: delta 5 desaturase; D6D: delta 6 desaturase; SFA: saturated fatty acid; MUFA: monounsaturated fatty acid; %E: percentage of total energy intake; PUFA: polyunsaturated fatty acid; LC-PUFA: long chain polyunsaturated fatty acid; RR: relative risk; CI: confidence interval. * This study looked at T2DM as an exposure and an outcome.

## Supplementary Materials

The following are available online at http://www.mdpi.com/2072-6643/10/6/758/s1, Table S1: Quality Assessment.

## References

[1] American Diabetes Association. http://www.diabetes.org/diabetes-basics/diagnosis/?loc=db-slabnav.

[2] Beale E.G. (2013). Insulin signaling and insulin resistance. J. Investig. Med..

[3] Guo S. (2014). Insulin signaling, resistance, and the metabolic syndrome: Insights from mouse models to disease mechanism. J. Endocrinol..

[4] World Health Organization. http://www.who.int/diabetes/en/.

[5] Wu Y., Ding Y., Tanaka Y., Zhang W. (2014). Risk factors contributing to type 2 diabetes and recent advances in the treatment and prevention. Int. J. Med. Sci..

[6] Hivert M.F., Vassy J.L., Meigs J.B. (2014). Susceptibility to type 2 diabetes mellitus—From genes to prevention. Nat. Rev. Endocrinol..

[7] Knowler W.C., Barrett-Connor E., Folwer S.E., Hamman R.F., Lachin J.M., Walker E.A., Nathan D.M. (2002). Reduction in the incidence of type 2 diabetes with lifestyle intervention or metformin. N. Engl. J. Med..

[8] Tuomilehto J., Lindstrom J., Eriksson J.G., Valle T.T., Hämäläinen H., Ilanne-Parikka P., Keinänen-Kiukaanniemi S., Laakso M., Louheranta A., Rastas M. (2001). Prevention of type 2 diabetes mellitus by changes in lifestyle among subjects with impaired glucose tolerance. N. Engl. J. Med..

[9] Sears B., Perry M. (2015). The role of fatty acids in insulin resistance. Lipids Health Dis..

[10] Moloney F., Yeow T.P., Mullen A., Nolan J.J., Roche H.M. (2004). Conjugated linoleic acid supplementation, insulin sensitivity, and lipoprotein metabolism in patients with type 2 diabetes mellitus. Am. J. Clin. Nutr..

[11] Fritsche K.L. (2015). The science of fatty acids and inflammation. Adv. Nutr..

[12] Olalla L.M.S., Muniz F.J.S., Vaquero M.P. (2009). N-3 fatty acids in glucose metabolism and insulin sensitivity. Nutr. Hops..

[13] Lardinois C.K., Starich G.H. (1991). Polyunsaturated fats enhance peripheral glucose utilization in rats. J. Am. Coll. Nutr..

[14] Chen C., Yu X., Shao S. (2015). Effects of omega-3 fatty acid supplementation on glucose control and lipid levels in type 2 diabetes: A meta-analysis. PLoS ONE.

[15] Tapsell L.C., Neale E.P., Satija A., Hu F.B. (2016). Foods, nutrients and dietary patterns: Interconnections and implications for dietary guidelines. Adv. Nutr..

[16] Zhang M., Picard-Deland E., Marette A. (2013). Fish and marine omega-3 polyunsaturated fatty acid consumption and incidence of type 2 diabetes: A systematic review and meta-analysis. Int. J. Endocrinol..

[17] Calder P.C. (2012). Mechanism of action of (*n-*3) fatty acids. J. Nutr..

[18] Lattka E., Illig T., Koletzko B., Heinrich J. (2010). Genetic variants of the FADS1-FADS2 gene cluster as related to essential fatty acid metabolism. Curr. Opin. Lipidol..

[19] Merino D.M., Ma D.W.L., Mutch D.M. (2010). Genetic variation in lipid desaturases and its impact on the development of human disease. Lipids Health Dis..

[20] Schaeffer L., Gohike H., Muller M., Heid I.M., Palmer L.J., Kompauer I., Demmelmair H., Illig T., Koletzko B., Heinrich J. (2006). Common genetic variants of the FADS1 FADS2 gene cluster and their reconstructed haplotypes are associated with the fatty acid composition in phospholipids. Hum. Mol. Genet..

[21] Malerba G., Schaeffer L., Xumerle L., Klopp N., Trabetti E., Biscuola M., Cavallari U., Galavotti R., Martinelli N., Guarini P. (2008). SNPs of the FADS gene cluster are associated with polyunsaturated fatty acids in a cohort of patients with cardiovascular disease. Lipids.

[22] Xie L., Innis S.M. (2008). Genetic variants of the FADS1 FADS2 gene cluster are associated with altered (*n-*6) and (*n-*3) essential fatty acids in plasma and erythrocyte phospholipids in women during pregnancy and in breast milk during lactation. J. Nutr..

[23] Minihane A.M. (2016). Impact of the genotype on EPA and DHA status and responsiveness to increased intakes. Nutrients.

[24] Simpoulos A.P. (2010). Genetic variants in the metabolism of omega-6 and omega-3 fatty acids: Their role in the determination of nutritional requirements and chronic disease risk. Exp. Biol. Med..

[25] Barman M., Nilsson S., Naluai A.T., Sandin A., Wold A.E., Sandberg A.S. (2015). Single Nucleotide polymorphism in the FADS gene cluster but no ELOVL2 gene are associated with serum polyunsaturated fatty acid composition and development of allergy (in a Swedish Birth Cohort). Nutrients.

[26] Ralston J.C., Matravadia S., Garudio N., Holloway G.P., Mutch D.M. (2015). Polyunsaturated fatty acid regulation of adipocyte FADS1 and FADS2 expression and Function. Obesity.

[27] Sholtz S.A., Kerling E.H., Shaddy D.J., Li S., Thodosoff J.M., Colombo J., Carlson S.E. (2015). Docosahexaenoic acid (DHA) supplementation in pregnancy differentially modulates arachidonic acid and DHA status across FADS genotypes in pregnancy. Prostaglandins Leukot. Essent. Fat. Acids.

[28] Higgins J.P.T., Green S. (2011). Cochrane Handbook for Systematic Reviews of Interventions.

[29] Kim O.Y., Lim H.H., Yang L.I., Chae J.S., Lee J.H. (2011). Fatty acid desaturase (FADS) gene polymorphism and insulin resistance in association with serum phospholipid polyunsaturated fatty acid composition in healthy Korean men: Cross-sectional study. Nutr. Metab..

[30] Kroger J., Zietemann V., Enzenbach C., Weikert C., Jansen E.H.J.M., Doring F., Joost H.G., Boeing H., Schulze M.B. (2011). Erythrocyte membrane phospholipids fatty acids, desaturase activity, and dietary fatty acids in relation to risk of type 2 diabetes in the European Prospective Investigation into Cancer and Nutrition (EPIC)-Potsdam Study. Am. J. Clin. Nutr..

[31] Cormier H., Rudkowska I., Thifault E., Lemieux S., Couture P., Vohl M.C. (2013). Polymorphism in fatty acid desaturase (FADS) gene cluster: Effects on glycemic controls following an omega-3 polyunsaturated fatty acids (PUFA) supplementation. Genes.

[32] Takkunen M.J., Schwab U.S., Mello V.D.F., Eriksson J.G., Lindstrom J., Tuomilehto J., Uusitupa M.I.J. (2016). Longitudinal associations of serum fatty acid composition with type 2 diabetes risk and markers of insulin secretion and sensitivity in the Finish Diabetes Prevention Study. Eur. J. Nutr..

[33] Yao M., Li J., Xie T., He T., Fang L., Shi Y., Hou L., Lian K., Wang R., Jiang L. (2015). Polymorphism of rs174616 in the FADS1-FADS2 gene cluster is associated with a reduced risk of type 2 diabetes mellitus in northern Han Chinese people. Diabetes Res. Clin. Pract..

[34] Huang M.C., Chang W.T., Chang H.Y., Chung H.F., Chen F.P., Huang Y.F., Hsu C.C., Hwang S.J. (2017). FADS gene polymorphism, fatty acid desaturase activities, and HDL-C in type 2 diabetes. Int. J. Environ. Res. Public Health.

[35] Wu J.H.Y., Micha R., Imamura F., Pan A., Biggs M.L., Ajaz O., Dujousse L., Hu F.B., Mozaffarian D. (2012). Omega-3 fatty acids and incident type 2 diabetes: A systematic review and meta-analysis. Br. J. Nutr..

[36] Wallin A., Di Giuseppe D., Orsini N., Patel P.S., Forouhi N.G., Wolk A. (2012). Fish consumption, dietary long-chain *n-*3 fatty acids and risk of type 2 diabetes: Systematic review and meta-analysis of prospective studies. Diabetes Care.

