# Supplementary material for: FADS Polymorphism, Omega-3 Fatty Acids and Diabetes Risk: A Systematic Review
Source: Nutrients. 2018 Jun 13;10(6):758. doi: 10.3390/nu10060758 (PMC6024808; doi:10.3390/nu10060758)
Supplement: Supplementary file 1 [file nutrients-10-00758-s001.pdf]

Table 1. Quality assessment

Article 1: Kim et al, 2011

DOMAIN	DESCRIPTION	REVIEWER ASSESSMENT	REVIEWER COMMENTS
Selection bias<br>**Random Sequence Generation**	Participants were recruited from the Health Service Center or by newspaper announcement for health examinations	Low risk	
Selection bias<br>**Allocation Concealment**	Doesn't apply	-	Since it was a cross-sectional study, there was no need for allocation concealment
Reporting Bias<br>**Selective reporting**	Study protocol is available and all of the study's pre-specified (primary and secondary) outcomes (FADS polymorphism, IR, serum phospholipids) that are of interest in the review have been reported in the pre-specified way	Low risk	
Other bias<br>**Other sources of bias**	None	Low risk	
Performance bias<br>**Blinding (participants and personnel)**	Participants and personnel were not blinded, however the outcome doesn't seem to be affected by it since it is an observational study	Low risk	
Detection bias<br>**Blinding (outcome assessment)**	No blinding of outcome assessment, but the review authors judge that the outcome measurement is not likely to be influenced by lack of blinding	Low risk	
Attrition bias<br>**Incomplete outcome data**	No missing outcome data	Low risk	

Article 2: Kroger et al, 2011

DOMAIN	DESCRIPTION	REVIEWER ASSESSMENT	REVIEWER COMMENTS
Selection bias<br>**Random Sequence Generation**	A case-cohort within the EPIC-Potsdam study designed. Randomly selected individuals from all participants of the previous study, using appropriate statistics	Low risk	Did not stated which statistics, but said that the subsample was representative of the whole EPIC-Potsdam study
Selection bias<br>**Allocation Concealment**	Doesn't apply	-	Since it was a prospective cohort, there was no need for allocation concealment
Reporting Bias<br>**Selective reporting**	Study protocol is available and all of the study's pre-specified (primary and secondary) outcomes (Physician diagnostic T2DM, RBC FA, FADS genotype, desaturase activity, dietary FA intake) that are of interest in the review have been reported in the pre-specified way	Low risk	
Other bias<br>**Other sources of bias**	None	Low risk	
Performance bias<br>**Blinding (participants and personnel)**	Participants and personnel were not blinded, however the outcome doesn't seem to be affected by it since it is an observational study	Low risk	
Detection bias<br>**Blinding (outcome assessment)**	No blinding of outcome assessment, but the review authors judge that the outcome measurement is not likely to be influenced by lack of blinding	Low risk	
Attrition bias<br>**Incomplete outcome data**	No missing outcome data	Low risk	

Article 3: Cormier et al, 2013

DOMAIN	DESCRIPTION	REVIEWER ASSESSMENT	REVIEWER COMMENTS
Selection bias<br>**Random Sequence Generation**	Individuals were recruited through advertisements and electronic messages	Low risk	
Selection bias<br>**Allocation Concealment**	All participants received supplementation.	-	Since all participants received supplementation the investigators couldn't have biased the selection of subjects
Reporting Bias<br>**Selective reporting**	Study protocol is available and all of the study's pre-specified (primary and secondary) outcomes (FG, FI, HOMA-IS – in response to n-3 supplementation, FADS genotype) that are of interest in the review have been reported in the pre-specified way	Low risk	
Other bias<br>**Other sources of bias**	None	Low risk	
Performance bias<br>**Blinding (participants and personnel)**	Participants and personnel were not blinded, however the outcome doesn't seem to be affected by it since all were supplemented. Individuals were also not aware of their genotype information.	Low risk	
Detection bias<br>**Blinding (outcome assessment)**	No blinding of outcome assessment, but the review authors judge that the outcome measurement is not likely to be influenced by lack of blinding	Low risk	
Attrition bias<br>**Incomplete outcome data**	No missing outcome data	Low risk	

Article 4: Yao et al, 2015

DOMAIN	DESCRIPTION	REVIEWER ASSESSMENT	REVIEWER COMMENTS
Selection bias<br>**Random Sequence Generation**	Individuals were recruited through a routine check-up in a Chinese Hospital. All subjects who met the eligibility criteria were recruited (for healthy case subjects or T2DM individuals)	Low risk	
Selection bias<br>**Allocation Concealment**	All participants had to answer the same questions	-	Since all participants had to go under the same protocol, a biased selection of subjects is improbable
Reporting Bias<br>**Selective reporting**	Study protocol is available and all of the study's pre-specified (primary and secondary) outcomes (FG, FI, HOMA-IS – in response to n-3 supplementation, FADS genotype) that are of interest in the review have been reported in the pre-specified way	Low risk	
Other bias<br>**Other sources of bias**	None	Low risk	
Performance bias<br>**Blinding (participants and personnel)**	Participants and personnel were not blinded, however the outcome doesn't seem to be affected by it since they weren't receiving an intervention	Low risk	
Detection bias<br>**Blinding (outcome assessment)**	No blinding of outcome assessment, but the review authors judge that the outcome measurement is not likely to be influenced by lack of blinding	Low risk	
Attrition bias<br>**Incomplete outcome data**	No missing outcome data	Low risk	

Article 5: Takkunen et al, 2016

DOMAIN	DESCRIPTION	REVIEWER ASSESSMENT	REVIEWER COMMENTS
Selection bias<br>**Random Sequence Generation**	Individuals who were at high risk for T2DM were recruited (from advertisement, epidemiological surveys, population screening) and randomized into a control or intensive lifestyle intervention group	Low risk	
Selection bias<br>**Allocation Concealment**	Not enough information:<br>- Did the people in each group know they were in case or control group? Or did they think they were all receiving the same information?	Unclear risk	
Reporting Bias<br>**Selective reporting**	Study protocol is available and all of the study's pre-specified (primary and secondary) outcomes (Serum FA composition, T2DM incidence, Insulin Secretion, Insulin sensitivity and disposition index) that are of interest in the review have been reported in the pre-specified way	Low risk	
Other bias<br>**Other sources of bias**	None	Low risk	
Performance bias<br>**Blinding (participants and personnel)**	Not enough information:<br>- Were the individuals aware that they were receiving different intervention?	Unclear risk	
Detection bias<br>**Blinding (outcome assessment)**	No information regarding blinding of outcome assessment	Unclear risk	
Attrition bias<br>**Incomplete outcome data**	No missing outcome data	Low risk	
